# Automatic segmentation and quantitative analysis of brain CT volume in 2-year-olds using deep learning model

**DOI:** 10.3389/fneur.2025.1573060

**Published:** 2025-04-24

**Authors:** Fengjun Xi, Liyun Tu, Feng Zhou, Yanjie Zhou, Jun Ma, Yun Peng

**Affiliations:** ^1^Department of Radiology, Beijing Tiantan Hospital, Capital Medical University, Beijing, China; ^2^School of Artificial Intelligence, Beijing University of Posts and Telecommunications, Beijing, China; ^3^Imaging Center, Beijing Children’s Hospital, National Center for Children’s Health, Capital Medical University, Beijing, China

**Keywords:** brain volume, deep learning, segmentation, CT, children

## Abstract

**Objective:**

Our research aims to develop an automated method for segmenting brain CT images in healthy 2-year-old children using the ResU-Net deep learning model. Building on this model, we aim to quantify the volumes of specific brain regions and establish a normative reference database for clinical and research applications.

**Methods:**

In this retrospective study, we included 1,487 head CT scans of 2-year-old children showing normal radiological findings, which were divided into training (*n* = 1,041) and testing (*n* = 446) sets. We preprocessed the Brain CT images by resampling, intensity normalization, and skull stripping. Then, we trained the ResU-Net model on the training set and validated it on the testing set. In addition, we compared the performance of the ResU-Net model with different kernel sizes (3 × 3 × 3 and 1 × 3 × 3 convolution kernels) against the baseline model, which was the standard 3D U-Net. The performance of the model was evaluated using the Dice similarity score. Once the segmentation model was established, we derived the regional volume parameters. We then conducted statistical analyses to evaluate differences in brain volumes by sex and hemisphere, and performed a Spearman correlation analysis to assess the relationship between brain volume and age.

**Results:**

The ResU-Net model we proposed achieved a Dice coefficient of 0.94 for the training set and 0.96 for the testing set, demonstrating robust segmentation performance. When comparing different models, ResU-Net (3,3,3) model achieved the highest Dice coefficient of 0.96 in the testing set, followed by ResU-Net (1,3,3) model with 0.92, and the baseline 3D U-Net with 0.88. Statistical analysis showed that the brain volume of males was significantly larger than that of females in all brain regions (*p* < 0.05), and age was positively correlated with the volume of each brain region. In addition, specific structural asymmetries were observed between the right and left hemispheres.

**Conclusion:**

This study highlights the effectiveness of deep learning for automatic brain segmentation in pediatric CT imaging, providing a reliable reference for normative brain volumes in 2-year-old children. The findings may serve as a benchmark for clinical assessment and research, complementing existing MRI-based reference data and addressing the need for accessible, population-based standards in pediatric neuroimaging.

## 1 Introduction

Abnormalities in brain volumetrics have been associated with congenital and acquired diseases in children, such as hydrocephalus (1), brain trauma (2), and neuropsychiatric disorders (3). Most *in vivo* studies have used magnetic resonance imaging (MRI) of healthy volunteers to measure global and regional volume loss (4–7). However, this approach encounters difficulties when applied to pediatric populations, resulting in a lack of an accepted normative database, which limits quantitative reporting (4, 5, 8).

CT is a fast, cost-effective, and widely accessible imaging modality, providing a viable alternative for pediatric patients unable to undergo MR examination, enabling the generation of a large reference database for statistical analysis (9, 10). Research indicates that CT-based visual classifications and quantitative metrics are comparable to MRI results for certain pathological features and show significant correlations with cognitive test outcomes (11–13). However, MRI is widely used for high-resolution brain volume measurements due to its strong soft tissue contrast. However, MRI remains the preferred modality for high-resolution brain volume measurements due to its superior soft tissue contrast. Previous CT volume assessments have primarily used semi-quantitative methods, which are time-intensive and require trained specialists. Recent advances in automated brain CT segmentation, particularly through deep learning, have demonstrated faster and more accurate segmentation, with results strongly correlating with those from MRI segmentation (14–16).

In this study, we focused on children aged 2 years, applying an automated segmentation algorithm to a large set of retrospectively identified head multidetector computed tomography (MDCT) scans with normal radiological findings to develop a clinical reference database for regional brain volumes. This database can serve as a quantitative benchmark for evaluating cases within similar clinical peer groups.

## 2 Materials and methods

### 2.1 Study cohort and imaging protocol

This study was a retrospective analysis of head CTs identified from the clinical PACS, with institutional review board approval and a consent waiver obtained prior to data collection.

We consecutively collected scans from a CT scanner between October 2017 and May 2022. Cases consisted of patients with nonspecific symptoms (e.g., head injury, headache, fever, vomiting) and no known systemic disease. All control cases were reviewed by two board-certified neuroradiologists and confirmed as normal, without acute or chronic abnormalities. Cases with image artifacts or a history of brain conditions-such as intracranial hemorrhage, skull fracture, or neurodevelopmental impairments-were excluded. Additionally, patients with prior or follow-up CT/MRI revealing intracranial abnormalities (e.g., cysts, hyperintense FLAIR lesions) were excluded from the analysis.

### 2.2 CT segmentation with ResU-Net model

In this study, we applied the ResU-Net (17) model to segment brain anatomical regions on CT images. First, this study involved 1,487 patients with multidetector computed tomography (MDCT) scans. We trained and tested the ResU-Net model on this dataset. Then, we used the trained ResU-Net model to obtain segmentation results on the CT data set. Further details on data acquisition, preprocessing, and model training and testing methodologies are provided below.

#### 2.2.1 Data acquisition

To train the ResU-Net model, 1,487 patients were enrolled. All enrolled patients underwent multidetector computed tomography (MDCT) scans. MDCT images were used for the brain segmentation task and volume analysis task. All scans were performed using a 256-row detector CT scanner (Revolution CT, GE Healthcare) in axial scan mode. The detector coverage was adjusted based on the patient’s head size, with options of 12, 14, or 16 cm. The tube voltage was set at 120 kVp, and the gantry rotation time was 0.8 s. The tube current was tailored to the children’s age, ranging from 150 mA for children aged 0–2 years, 170 mA for children aged 3–6 years, 190 mA for children aged 7–12 years, and 210 mA for children aged 13 years and older. Additionally, radiologic technologists could adjust the tube current by ±10 mA based on their experience.

The scan matrix size was 512 × 512, with both slice thickness and slice spacing set to 0.625 mm. The volume CT dose index (CTDI_VOL_) was approximately 16–20 mGy. The original scan data were then reconstructed into standard window images with a slice thickness of 0.625 mm and a slice spacing of 0.625 mm. Bone window images were also reconstructed using a window width of 4,000 and a window level of 700, while standard window images had a window width of 100 and a window level of 30. These images were subsequently uploaded to the PACS system.

The scans were performed in a single rotation with the patient in a fixed, supine position. The scanning range extended from the base of the skull to the top of the skull. For children unable to cooperate during the procedure, sedation was administered using oral chloral hydrate (10%, 0.4 mL/kg) before the scan.

The dataset was divided into training (*n* = 1,041) and test (*n* = 446) sets in a 7:3 ratio. The training set was utilized to train the ResU-Net model, while the test set was reserved for independent evaluation to assess the model’s performance. This approach ensures that the model is trained on a sufficiently large sample while also enabling an unbiased assessment of its ability to generalize to new, unseen data.

#### 2.2.2 Data preprocessing

Before training and testing the model, we applied a series of preprocessing steps to all the images, including resampling, intensity normalization, and skull stripping. First, we resampled all 3D images using linear interpolation to achieve a voxel spacing of 1 × 1 × 1 mm^3^. Next, we performed intensity normalization through adaptive histogram equalization to enhance the contrast of the images. Finally, we used the Python library SimpleITK to perform skull stripping by applying threshold segmentation to remove the skull. This process eliminated the skull’s occlusion of the brain tissue, allowing for clearer visualization of the brain tissue’s morphology and density.

In this study, expert labeling was first established through manual labeling by a neuroradiologist with 6 years of experience, under the supervision of a pediatric neurodiagnostic specialist with over 15 years of expertise. Segmentation followed neuroanatomical atlases and previous studies (16, 18–21), with manual labeling performed on axial slices and adjusted in coronal and sagittal views. These expert labels were then used as the segmentation templates. To generate a large labeled dataset for model training, pseudo-labels were created through a template-based registration process using ANTs (Advanced Normalization Tools). The pseudo-labels were then roughly visually inspected by the experts to correct any evident segmentation errors. After correction, these pseudo-labels were treated as the ground truth labels for training the deep learning model.

The segmentation focused on 10 brain regions, including the right/left frontal lobes, parietal lobes, occipital lobes, temporal lobes, cerebellum, and brainstem. These regions were selected based on their clear anatomical boundaries and the feasibility of accurate segmentation in pediatric CT images. Due to the ongoing myelination process in children’s brains, distinguishing gray and white matter is challenging. As a result, we prioritized larger brain lobes and structures that are more easily identifiable in CT scans. Manual labeling was performed on axial slices, with adjustments made in coronal and sagittal views to ensure accuracy. ITK-SNAP software (Version 3.6.0) was used for these manual annotations.

#### 2.2.3 Model training and testing

**Figure 1:**
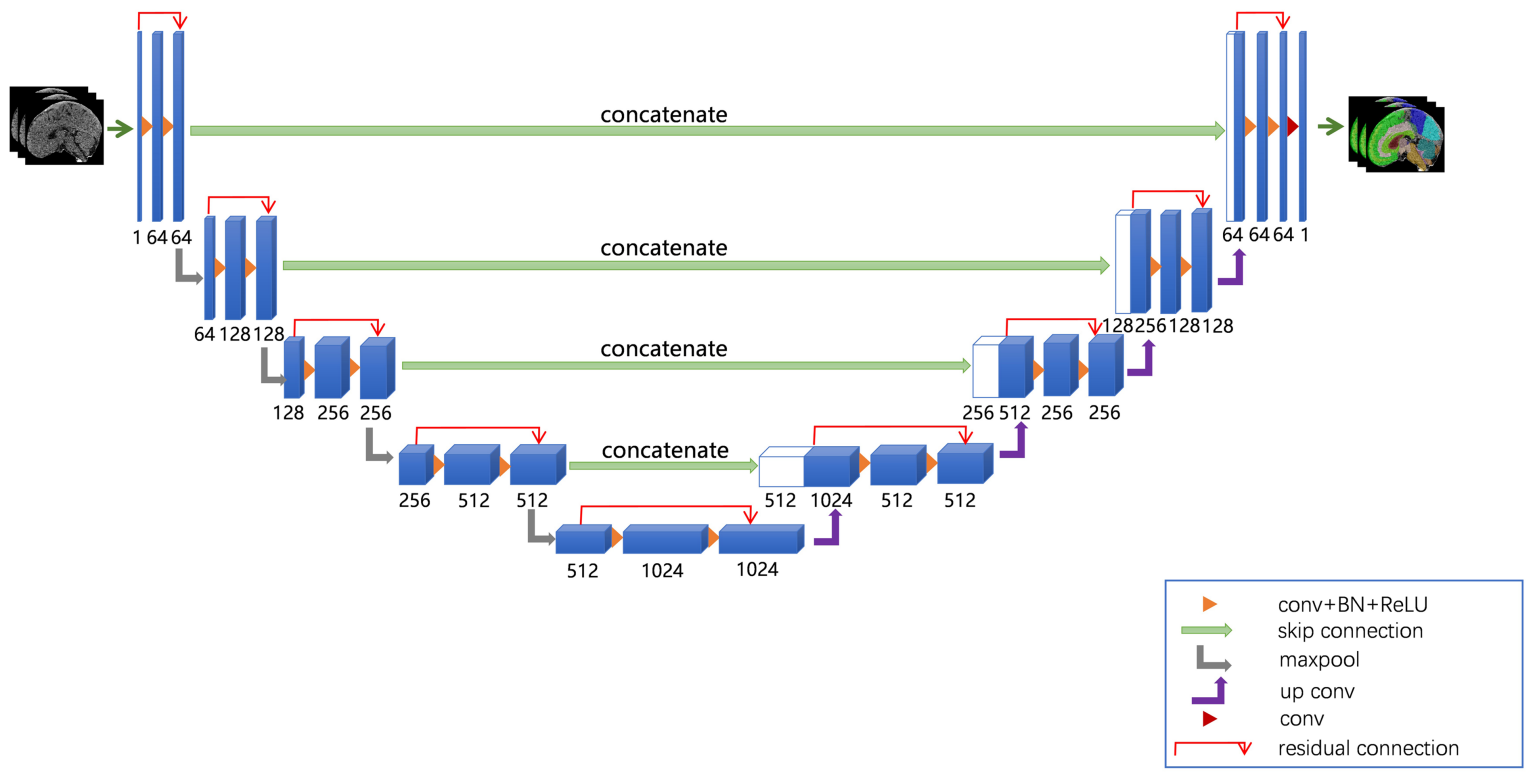
ResU-Net used in this study. The ResU-Net network processed 3D CT images through a combination of residual connections, U-Net skip connections, sub-sampling, and up-sampling operations.

The network architecture of the ResU-Net model used in this study is shown in Figure 1. The ResU-Net model processes 3D CT images through a combination of residual connections, U-Net skip connections, subsampling, and up-sampling operations. It uses 27 convolutional layers to achieve precise and detailed segmentation, enhancing convergence speed and computational efficiency.

We employed the ResU-Net model to segment brain structures. To enhance the robustness of the model, we selected data augmentation methods, including random flips and rotations, for the training data, while no augmentation was applied to the testing data to ensure a fair evaluation of the model’s performance. Next, we built the deep learning model in PyTorch and trained it with five-fold cross-validation. Model parameters were updated using a multi-class cross-entropy loss function and the Adam optimizer, with a learning rate of 1 × 10^−5^. Training was conducted over 200 epochs with a batch size of 4. Finally, the model’s performance was evaluated using the Dice similarity score (22).

#### 2.2.4 Comparison of ResU-Net configurations

In this study, we compared the performance of ResU-Net network with different kernel sizes by evaluating three configurations: ResU-Net model with a 3 × 3 × 3 convolution kernel, ResU-Net model with a 1 × 3 × 3 convolution kernel, and the baseline model, which was the standard 3D U-Net model. To assess segmentation accuracy, we calculated the average Dice coefficient over 10 training sessions to compare the performance of the different models. These results were analyzed to evaluate the impact of varying kernel sizes on model performance, with the baseline 3D U-Net serving as a reference for comparing the improvements or trade-offs introduced by modifying the kernel size in ResU-Net network.

### 2.3 Statistical analysis

All statistical analyses were performed on SPSS 26.0 software. Descriptive statistics are reported as mean ± standard deviation. We conducted one-sample t-tests and analysis of variance (ANOVA) to examine the age and sex distributions of the samples in both the training and testing sets, ensuring that no bias was introduced in the group assignments. Next, we assessed the effects of sex and age on brain structure volumes using multiple linear regression analyses. Specifically, sex was included as a categorical variable (coded as 1 for males and 2 for females) in the regression models to isolate its impact on brain volume, while also adjusting for age. Additionally, a paired t-test was used to compare the left and right hemispheres, with the corresponding *p*-value calculated to evaluate the statistical significance of the difference. A *p* value of < 0.05 was considered statistically significant.

## 3 Results

### 3.1 Performance of ResU-Net model during training and testing

**Figure 2:**
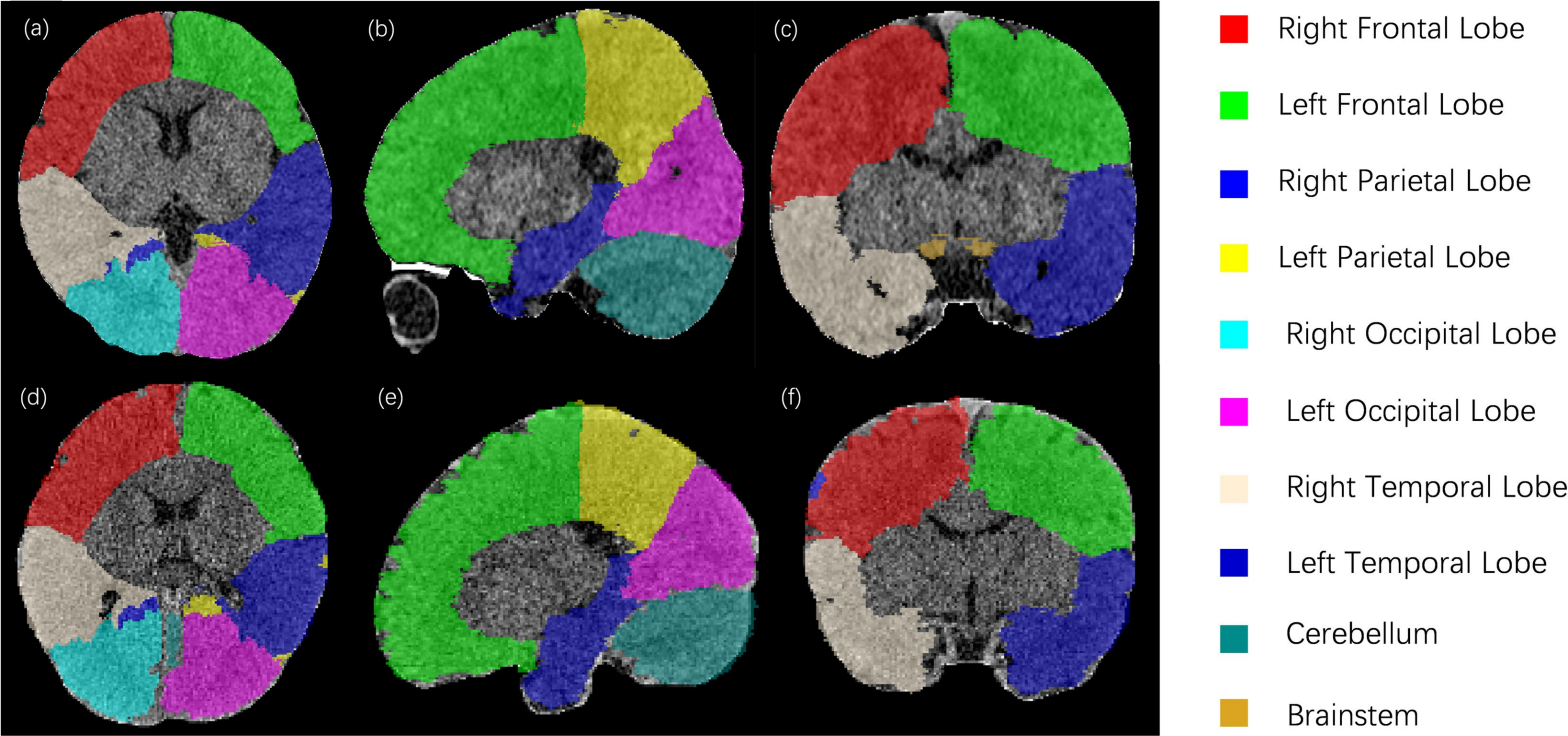
Segmentation results for a sample subject from the testing set. Panels **(a–c)** show axial, sagittal, and coronal CT sections with ground truth labels, while panels **(d–f)** display the corresponding sections with labels generated by the ResU-Net model. Ten brain regions—right and left frontal lobes, right and left parietal lobes, right and left occipital lobes, right and left temporal lobes, cerebellum, and brainstem—are color-coded and labeled on the right.

Overall, the ResU-Net model demonstrated strong performance in segmenting intracranial brain tissue, achieving an average Dice coefficient of 0.94 on the training set and 0.96 on the test set. Figure 2 presents representative segmentation results for 10 brain anatomical regions from a sample in the testing set. Figures 2a–c display axial, sagittal, and coronal sections with ground truth segmentation labels, while Figures 2d–f show the corresponding sections with ResU-Net model generated labels. Overall, our model results aligned closely with the ground truth, although some minor missed and extra labels appear at the edges in Figures 2d,e.

### 3.2 Comparison of model performance

In the training set, the Dice coefficients for the Baseline model, ResU-Net (1,3,3) model, and ResU-Net (3,3,3) models were 0.94, 0.93, and 0.92, respectively. These values indicate similar performance in segmentation accuracy, with the Baseline model slightly outperforming the ResU-Net configurations. The small differences in training Dice coefficients suggest that all models effectively learned to segment brain regions.

However, in the test set, the ResU-Net (1,3,3) models and ResU-Net (3,3,3) models showed substantial improvements, achieving Dice coefficients of 0.92 and 0.96, respectively, while the Baseline model dropped to 0.88. This indicates that the ResU-Net models, particularly the ResU-Net (3,3,3) model, generalize better to unseen data. This is likely due to their ability to capture more robust features and adapt to variations in test images more effectively than the Baseline model.

The decline in the Baseline model’s performance from training (0.94) to testing (0.88) suggests overfitting to the training data. This reinforces the advantages of using ResU-Net network with residual connections, as these models maintained consistent performance across both training and testing, thereby reducing the risk of overfitting and improving the model’s generalization ability.

### 3.3 Sample demographics

The cohort included 826 males and 661 females, totaling 1,487 participants. The ages of the participants ranged from 1.5 to 2.5 years, with a mean age of 1.98 ± 0.28 years. There were no statistically significant differences in age or gender distribution between the training and test sets (*p* > 0.05). Further details are provided in Table 1.

**Table 1:** Demographics characteristics between training and testing set.

Group	Age (years)	Sex (*n*)	
		Male	Female
Training set (*n* = 1,074)	1.97 ± 0.28	603	471
Testing set (*n* = 413)	1.98 ± 0.28	223	190
*p* value	0.554	0.491	

This table shows the demographic characteristics of the training and testing sets, including age and sex distribution. The age of the two groups was 1.97 ± 0.28 and 1.98 ± 0.28, respectively, and there was no significant difference in age distribution (*p* = 0.554). In addition, there was no statistically significant difference in gender distribution between the training and test groups (*p* = 0.491).

### 3.4 Influence of sex and age on brain volume

The average brain region volumes in healthy 2-year-old children were as follows: right frontal lobe (200.64 ± 19.15 cm^3^), left frontal lobe (204.51 ± 19.61 cm^3^), right parietal lobe (81.52 ± 7.91 cm^3^), left parietal lobe (79.42 ± 7.65 cm^3^), right occipital lobe (53.76 ± 5.21 cm^3^), left occipital lobe (52.82 ± 5.33 cm^3^), right temporal lobe (91.92 ± 9.07 cm^3^), left temporal lobe (94.08 ± 9.27 cm^3^). Additionally, the cerebellum volume was 127.97 ± 11.85 cm^3^, and the brainstem volume was 15.47 ± 1.50 cm^3^. A more detailed description is provided in Table 2, which includes the distribution of brain volumes by sex.

**Table 2:** Distribution of brain structure volumes (cm^3^).

Brain regions	Overall	Male	Female
Right frontal lobe	200.64 ± 19.15	208.01 ± 18.51	191.43 ± 15.63
Left frontal lobe	204.51 ± 19.61	211.90 ± 18.99	195.28 ± 16.15
Right parietal lobe	81.52 ± 7.91	84.61 ± 7.51	77.66 ± 6.60
Left parietal lobe	79.42 ± 7.65	82.35 ± 7.31	75.76 ± 6.41
Right occipital lobe	53.76 ± 5.21	55.53 ± 5.02	51.55 ± 4.55
Left occipital lobe	52.82 ± 5.33	54.45 ± 5.25	50.78 ± 4.69
Right temporal lobe	91.92 ± 9.07	95.55 ± 8.66	87.40 ± 7.38
Left temporal lobe	94.08 ± 9.27	97.85 ± 8.81	89.38 ± 7.53
Cerebellum	127.97 ± 11.85	132.56 ± 11.36	122.23 ± 9.78
Brainstem	15.47 ± 1.50	16.04 ± 1.46	14.75 ± 1.21

This table shows the brain volume distribution in detail for the overall, male group, and female group. It shows the volumes (in cm^3^) of different brain regions including the frontal, parietal, occipital, and temporal lobes as well as the cerebellum and brainstem.

We analyzed the effect of age and sex on brain volume using multiple regression analysis. The results in Table 3 demonstrate the effect of gender on volume. Statistical analysis showed that the brain volume of males was generally larger than that of females at the same age, and the results were statistically significant (*p* < 0.001).

**Table 3:** Effect of sex on brain volume.

Brain region	Sex coefficient (B)	Standard error (std. error)	t-value	*p*-value
Right frontal lobe	−15.282	0.851	−17.95	*p* < 0.001
Left frontal lobe	−15.356	0.882	−17.418	*p* < 0.001
Right parietal lobe	−6.482	0.356	−18.215	*p* < 0.001
Left parietal lobe	−6.108	0.344	−17.742	*p* < 0.001
Right occipital lobe	−3.743	0.246	−15.193	*p* < 0.001
Left occipital lobe	−3.464	0.258	−13.423	*p* < 0.001
Right temporal lobe	−7.622	0.406	−18.763	*p* < 0.001
Left temporal lobe	−7.962	0.416	−19.15	*p* < 0.001
Cerebellum	−9.766	0.544	−17.955	*p* < 0.001
Brainstem	−1.201	0.068	−17.745	*p* < 0.001

This table presents the effect of gender on brain volume in the multiple regression analysis. The results showed that the gender coefficients were all negative, indicating that the brain volume of the male group was generally larger than that of the female group in all brain regions (*p* < 0.001).

**Figure 3:**
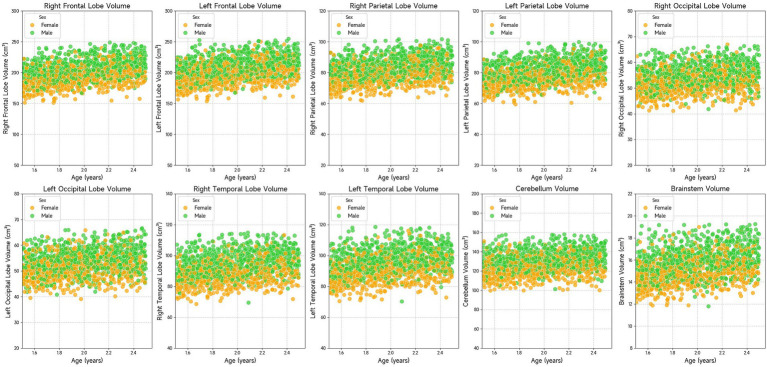
Scatter plots showing the volume of various brain regions (right frontal lobe, left frontal lobe, right parietal lobe, left parietal lobe, right occipital lobe, left occipital lobe, right temporal lobe, left temporal lobe, cerebellum, and brainstem) in relation to age. The data suggests that brain region volumes generally increase with age. Males and females are represented with green and orange dots, respectively.

The results in Table 4 present the effect of age on brain volume. Statistical results showed that age had a significant effect on each brain volume (*p* < 0.001). Brain volume increases with age in all brain regions. However, brain volumes in different regions grew at different rates because they had different regression coefficients. Figure 3 visualizes the relationship between age and brain volumes, showing how the volumes of these regions increase with age.

**Table 4:** Effect of age on brain volume.

Brain region	Age coefficient (B)	Standard error (std. error)	t-value	*p*-value
Right frontal lobe	21.179	1.488	14.236	*p* < 0.001
Left frontal lobe	20.714	1.541	13.446	*p* < 0.001
Right parietal lobe	7.691	0.622	12.37	*p* < 0.001
Left parietal lobe	7.833	0.602	13.021	*p* < 0.001
Right occipital lobe	3.884	0.43	9.021	*p* < 0.001
Left occipital lobe	3.39	0.451	7.518	*p* < 0.001
Right temporal lobe	8.642	0.71	12.174	*p* < 0.001
Left temporal lobe	8.359	0.726	11.507	*p* < 0.001
Cerebellum	9.264	0.95	9.747	*p* < 0.001
Brainstem	1.45	0.118	12.262	*p* < 0.001

This table shows the effect of gender on brain volume in the multiple regression analysis. The results showed that the coefficients of gender were all positive, indicating that there was a positive correlation between age and brain volume, that is, brain volume increased with age (*p* < 0.001). And the results show that the change rate of brain volume in different regions is not consistent because of the different coefficient values.

### 3.5 Comparison of brain volumes by hemisphere

We performed paired comparisons between the left and right sides of symmetrical structures in the brain, including frontal, parietal, occipital, and temporal lobes. The statistical results in Table 5 show that there are statistical differences in the left and right brain volumes of each lobe (*p* < 0.001), which indicates the asymmetry of the left and right sides.

**Table 5:** Comparison of left and right brain structure volumes.

Brain regions	Sex	Right	Left	t value	Effect size	*p*-value
Frontal lobe	M	208.01 ± 8.51	211.90 ± 8.99	−30.54	1.06	<0.001
	F	191.43 ± 5.63	195.28 ± 6.15	−29.60	1.15	<0.001
Parietal lobe	M	84.61 ± 7.51	82.35 ± 7.31	26.72	0.93	<0.001
	F	77.66 ± 6.60	75.76 ± 6.41	22.39	0.87	<0.001
Occipital lobe	M	55.53 ± 5.02	54.45 ± 5.25	11.60	0.40	<0.001
	F	51.55 ± 4.55	50.78 ± 4.69	8.13	0.32	<0.001
Temporal lobe	M	95.55 ± 8.66	97.85 ± 8.81	−24.53	0.85	<0.001
	F	87.40 ± 7.38	89.38 ± 7.53	−19.34	0.75	<0.001

This table compares the brain structure volumes between the left and right hemispheres for both males and females. It includes the frontal, parietal, occipital, and temporal lobes. Statistically significant differences are observed in all regions, with the right hemisphere showing larger volumes in the frontal and parietal lobes, and the left hemisphere having larger volumes in the occipital and temporal lobes. Effect sizes are moderate to high, reflecting the magnitude of the differences.

## 4 Discussion

Childhood is a critical period for brain development, particularly in the first few years of life when the brain undergoes rapid growth and completes many developmental stages (6, 23). Brain development is influenced by various internal and environmental factors (24), and diseases during this period can significantly impact development (3, 7, 24–26). Brain volume is a key measure of brain development, obtained through non-invasive neuroimaging techniques such as high-resolution MRI, and is an important indicator of health and disease status in children. Quantitative assessments of brain volume are increasingly used in studies of neurological disorders (25, 27, 28). However, the lack of population-based reference standards for healthy brain volume complicates the clinical assessment of individual diseases (29, 30). Due to the limited availability of high-resolution MRI data in children and the small sample sizes in related studies, establishing a normative reference standard for brain volume remains challenging (4, 5, 31, 32). In addition, MRI may not be feasible in certain clinical scenarios, such as emergency situations or with patients who have medical devices that cause significant artifacts (e.g., endotracheal tubes or other hardware). These limitations can restrict the ability to obtain reliable brain volume measurements in diverse pediatric populations.

CT imaging, with its faster acquisition time and wider clinical application, offers an opportunity to accumulate large datasets, making it feasible for studies involving larger and more diverse populations. In this study, CT data were used to measure brain volumes, with the expectation that these findings will complement and extend results from MRI studies. By leveraging the strengths of CT imaging, we aim to address some of the limitations associated with MRI and provide a more comprehensive understanding of brain development and disease in children.

This study utilized the ResU-Net deep learning model for brain tissue segmentation. In recent years, deep learning has become increasingly prevalent in medical image processing, particularly in segmentation tasks, due to its speed and robustness (33–39). Previous research has shown that deep learning algorithms perform well in brain tissue segmentation, with results comparable to those from MRI (14, 16). The Dice coefficient of 0.96 obtained in this study demonstrates that the ResU-Net algorithm achieved good segmentation efficiency. Furthermore, the use of residual connections in ResU-Net model enhances feature propagation and helps mitigate the risk of overfitting, which is particularly crucial when working with complex and noisy medical imaging data. This advantage is supported by other studies, which have demonstrated that incorporating residual connections into network architectures leads to improvements in segmentation accuracy (40).

Our findings indicate that brain volume in males is generally larger than in females, which is consistent with previous studies (5, 7, 41, 42). Research suggests that male brain volume is larger than that of females across all ages (41, 43). This difference may be influenced by several factors, including gonadal hormones, neurosteroids, and epigenetic and environmental factors (44–47). These influences contribute to significant sex-based differences in brain development, which may also affect the manifestation of neuropsychiatric disorders, neurodegenerative diseases, and trauma-related conditions (44).

Brain volume development varies across regions and ages during childhood, which is essential for understanding cognitive and neurological development. The brain grows rapidly during childhood, with different regions maturing at different rates (6, 48, 49). Gray matter volume peaks around age 6 before gradually decreasing, whereas white matter continues to develop until approximately age 28. The growth trajectories of different regions are heterogeneous, with regions associated with sensory and motor control reaching developmental peaks earlier, while prefrontal regions related to higher cognitive functions develop more slowly. Although this study focused on 2-year-olds, we observed a positive correlation between brain region volume and age, confirming the rapid brain development occurring at this age.

Moreover, the study also revealed structural asymmetry in the brain: the left frontal and temporal lobes were larger than their right counterparts, while the right parietal and occipital lobes were larger than the left. Although the brain exhibits a high degree of left–right symmetry at a macroscopic anatomical and functional level, subtle structural differences exist between hemispheres (50). This asymmetry is closely linked to higher cognitive functions such as language processing, spatial cognition, facial recognition, and emotional response (51). For example, the left hemisphere is more involved in language production and comprehension, while the right hemisphere plays a key role in processing non-verbal information. Brain asymmetry is influenced by factors such as genetics, environment, and hormones (50). The study of brain lateralization is crucial for understanding brain mechanisms and identifying specific diseases. Previous studies have shown that changes in brain asymmetry are associated with neurodegenerative diseases and neuropsychiatric disorders (52, 53). These findings may provide useful biomarkers or clinical predictors of disease, offering insights into the neurobiology of these conditions.

Several factors should be considered when interpreting these results. One challenge is the reliance on a single manual segmentation template for brain CT data, particularly due to the lack of standardized templates for children. This reliance may affect the accuracy and generalizability of the findings. Therefore, future research should focus on developing multiple segmentation templates to enhance both accuracy and model robustness. Another limitation is that the data in this study were obtained from a single center and a single device, with no validation from multi-center or multi-device datasets. This could impact the model’s adaptability to different scanning devices, scanning parameters, and image qualities. For instance, in real clinical settings, imaging data may be affected by noise, artifacts, or inadequate resolution. The robustness of the model in this study to such low-quality data has not been thoroughly tested, which could impact its reliability in practical applications. To address this issue, future studies will aim to expand data sources through multi-center collaborations and establish standardized data-sharing platforms to foster data exchange and promote collaborative research across various studies. Moreover, while this study focused on a “normal” population, the sample primarily consisted of trauma patients. Although efforts were made to exclude abnormal cases, minor trauma that was not identified by CT scans may have been overlooked, potentially affecting brain volume measurements. To improve detection of minor trauma, future research will develop deep learning-based detection techniques. Furthermore, collecting more detailed clinical information in subsequent studies will help to exclude such cases, ensuring that the findings are both accurate and comparable across different cohorts.

In conclusion, this study highlights the potential of deep learning for automatic brain tissue segmentation. Using existing clinical head CT data, our approach lays the groundwork for future clinical applications in diagnosis, treatment, and research.

## Data Availability

The original contributions presented in the study are included in the article, further inquiries can be directed to the corresponding authors.
